# Supplementary material for: Luminance effects on pupil dilation in speech-in-noise recognition
Source: PLoS One. 2022 Dec 2;17(12):e0278506. doi: 10.1371/journal.pone.0278506 (PMC9718387; doi:10.1371/journal.pone.0278506)
Supplement: S1 Appendix — (DOCX) [file pone.0278506.s001.docx]

**S1 Appendix**

There were 20 sentences and hence 20 pupil recordings per list. Standard methods of analysis [33] follow a block-based approach, where all traces for a given condition in a given subject are first aggregated together before proceeding to the extraction of the PPD. Being smoother and more stereotypical, such averaged traces are easier to deal with, but they hide variance associated with different speech materials and noise sources intrinsic to the listener’s cognitive availability. Here, and because we found this approach to be important in some cases [18], we reiterated all our analyses on a trial basis to explore whether new insights could be gleaned from the role of SNR, luminance, and position within a list.

**Experiment 1**

We considered a model with one fixed factor (*SNR*) and one random factor (*subject*), including both intercepts and slopes as they were now up to 20 observations per condition. Thus the model was of the form: “Intelligibility ~ SNR + (1+SNR | subject)”. It revealed a main effect of SNR [χ^2^(1)=16.5, p<0.001], with a lower performance at 0 dB than any other SNR (p<0.001). We then added *position* as a second fixed factor (treated as a continuous variable, and keeping by-*subject* random intercepts and slopes). There was no main effect of position [χ^2^(1)=2.1, p=0.143], but a weak interaction with SNR [χ^2^(3)=8.8, p=0.032]. This interaction was not very meaningful as it was largely driven by inhomogeneity of variance: performance was more variable across trials at 0 dB than at favorable SNRs (since performance was homogeneously closer to ceiling). Linear regressions were conducted at each SNR and failed to reach significance in any case [p>0.123, not plotted], and still missed significance with RAU-transformed data [p>0.085]. Thus, it did not point to any fatigue or practice effect. Consequently, we did not grant much value to this interaction.

The three metrics of pupil data were extracted from individual traces (rather than block-averaged) and examined through this more complex model of the form: “pupil metric ~ SNR + (1+SNR | subject)”.

For *baseline*: there was no main effect of SNR [χ^2^(3)=6.2, p=0.101], replicating the finding in the article. Interestingly, adding *position* (as a continuous variable) led to a main effect [χ^2^(1)=12.7, p<0.001] that interacted with SNR [χ^2^(3)=16.4, p<0.001]. As illustrated in Fig.S1 (top-left), the pupil baseline tended to decrease as trials progressed throughout the list, or at least this was true of the first 4-5 trials, and the slope of this effect differed somewhat across SNRs. Linear regressions showed that the +14 dB condition was the only one significant (p=0.008). Note that this relaxation phenomenon has been described before, and some groups have even proposed to discard the first few trials as not representative of the rest of the block [44].

For *PPD amplitude*: there was a main effect of SNR [χ^2^(3)=14.7, p=0.002], driven by larger PPDs at 0 dB than at any other SNR (p<0.001). Estimates ranged between 0.07 and 0.09 mm (Fig.S1, top-middle), replicating the finding in the article. Adding *position* did not lead to a main effect [χ^2^(1)=2.1, p=0.146] or an interaction [χ^2^(3)=1.8, p=0.608].

For *PPD latency*: there was a main effect of SNR [χ^2^(3)=8.6, p=0.035], driven by later PPDs at 0 dB than at any other SNR (p=0.002, p=0.005, and p=0.019 respectively for +7, +14, and quiet). This stands in contrast with the results mentioned in the article. Presumably, having 20 times more observations, this trial-based approach has more statistical power to reveal weaker effect sizes such as the finding of later PPD in more adverse listening situations (Fig.S1, top-right). Adding *position* did not lead to a main effect [χ^2^(1)=0.8, p=0.373] or interaction [χ^2^(3)=4.0, p=0.266].

Taken together, this trial-based analysis was largely consistent with the results mentioned in the article, but seems more sensitive to effects on PPD latency than the block-based approach and it allowed to reveal a trend for a pupil relaxation throughout the list.

**Experiment 2**

We considered a model with two fixed factors (*SNR* and *luminance*) and one random factor (*subject*), including both intercepts and slopes. Thus the model was of the form: “Intelligibility ~ SNR*luminance + (1+SNR*luminance | subject)”. It revealed a main effect of SNR [χ^2^(1)=42.3, p<0.001], but no main effect of luminance [χ^2^(2)=0.2, p=0.917] or interaction with SNR [χ^2^(2)=0.7, p=0.698]. We then added *position* as a third fixed factor (keeping by-*subject* random intercepts and slopes). There was no main effect of position [χ^2^(1)=1.5, p=0.227], no interaction between position and SNR [χ^2^(1)=1.6, p=0.202], no interaction between position and luminance [χ^2^(2)=2.8, p=0.244], and no 3-way interaction [χ^2^(2)=4.5, p=0.107]. Thus, position of a sentence within a list had no impact on its intelligibility, regardless of SNR and luminance, and this analysis did not add anything to the behavioral results described in the article.

The three metrics of pupil data were extracted from individual traces and examined through this more complex model of the form: “pupil metric ~ SNR*luminance + (1+SNR*luminance | subject)”.

For *baseline*: there was no main effect of SNR [χ^2^(1)=2.2, p=0.139], but a main effect of luminance [χ^2^(2)=64.9, p<0.001] without interaction [χ^2^(2)=4.7, p=0.095]. The results were thus in line with those reported in the main text. Interestingly, *position* led to a main effect [χ^2^(1)=26.2, p<0.001] and interacted with luminance [χ^2^(2)=12.5, p=0.002], but not with SNR [χ^2^(1)=0.2, p=0.658] or in a 3-way [χ^2^(2)=0.6, p=0.738]. The pupil baseline tended to decrease as trials progressed throughout the list (Fig.S1, bottom-left) and this was particularly the case in darkness (p<0.001). Linear regressions did not reach significance under medium or bright lights (p>0.100). This is curious because one could have expected the opposite phenomenon: even though we let a few minutes pass for the pupil to adjust before starting the recording in darkness, one could have expected a continuing dilation with prolonged time under darkness (given the sluggish nature of the dark-adaptation curve taking up to 20-30 minutes to reach a plateau [28]).

For *PPD amplitude*: there was no main effect of SNR [χ^2^(1)=1.6, p=0.211], but a main effect of luminance [χ^2^(2)=16.6, p<0.001] without interaction [χ^2^(2)=3.8, p=0.151]. Adding *position* as a third fixed factor led to a main effect [χ^2^(1)=12.2, p<0.001] that interacted slightly with SNR [χ^2^(1)=4.2, p=0.041], but not with luminance [χ^2^(2)=1.0, p=0.620] or in a 3-way [χ^2^(2)=0.8, p=0.667]. The PPD tended to soften throughout the list at 0 dB (p=0.015), but not at +14 dB (p=0.437). In other words, there were effects of SNR, consistently with the results reported in the article, but these effects were more apparent at the beginning of the lists.

For *PPD latency*: there was a main effect of SNR [χ^2^(1)=17.6, p<0.001], and a main effect of luminance [χ^2^(2)=10.2, p=0.006] without interaction [χ^2^(2)=1.4, p=0.493]. Adding *position* as a third fixed factor did not lead to a main effect [χ^2^(1)<0.1, p=0.860] but interacted slightly with SNR [χ^2^(1)=4.5, p=0.034], not with luminance [χ^2^(2)=1.2, p=0.535] or in a 3-way [χ^2^(2)=1.2, p=0.548]. The *position by SNR* interaction reflected that latency tended to increase throughout the list at 0 dB, but tended to decrease throughout the list at 14 dB. We did not grant much value to this interaction because none of the linear regressions ever reached significance for any condition, confirming that position played negligible role on PPD latency. These findings were largely consistent with those reported in the article but granted a role for luminance (later latency with darker luminance, Fig.S1 bottom-right) that was not revealed by a block-based analysis.


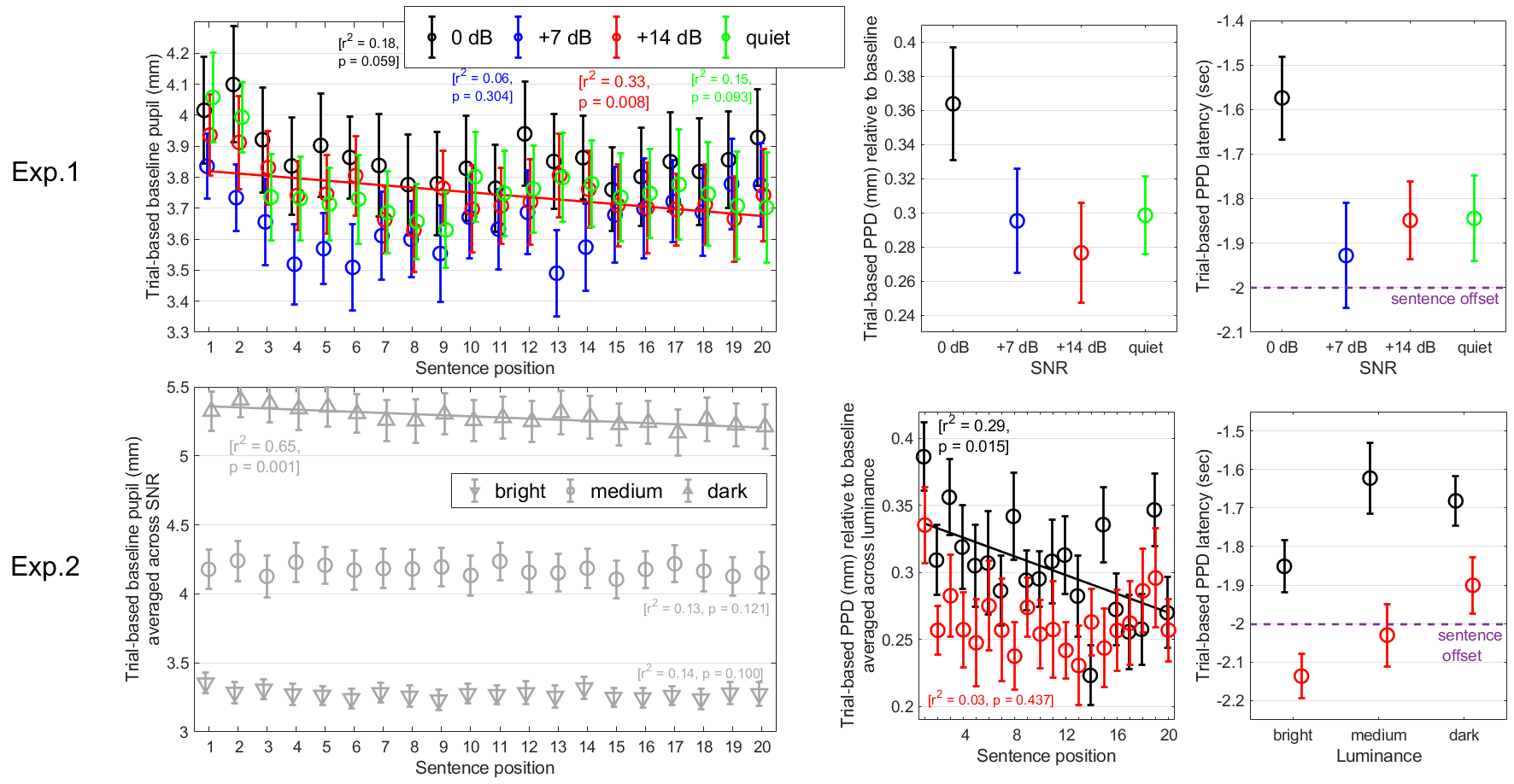


**FIG. S1 Performance in a sentence recognition task, with lists of twenty sentences presented at different SNRs**

To summarize, this trial-based approach confirmed the huge impact of luminance on baseline, and confirmed that PPD amplitude was underestimated in either bright or dark settings (but similarly across SNRs), but it added a new insight namely that PPD latency is likely delayed in darker testing environments. This approach could also reveal a small relaxation of the pupil baseline throughout the list, and the effect was exacerbated in dark settings.
