# Supplementary material for: Luminance effects on pupil dilation in speech-in-noise recognition
Source: PLoS One. 2022 Dec 2;17(12):e0278506. doi: 10.1371/journal.pone.0278506 (PMC9718387; doi:10.1371/journal.pone.0278506)
Supplement: S2 Appendix — (DOCX) [file pone.0278506.s002.docx]

**S2 Appendix**

Another finding consistently reported in the literature is the relationship to behavior, meaning here the difference in pupil dynamics when the sentence is correctly identified versus when it is misunderstood [44]. Here, we investigated 1) whether we could replicate this finding in typical luminance settings, and 2) whether this contrast could still be revealed in the dark or bright luminance settings since they appeared suboptimal in revealing PPDs. There were (as intended) few sentences that were misunderstood given that intelligibility was close to 90% at 0 dB SNR. To maximize their occurrence and make for a more balanced comparison, we categorized any sentence as “incorrect” when at least one word in the sentence was not identified and used this categorical variable as a fixed factor in LME analyses. Note that this approach relates to intelligibility relative to a given sentence, and therefore it is intrinsically a trial-based approach.

In Exp.1, for PPD amplitude, there was a main effect of *response type* [χ^2^(1)=4.9, p=0.027], but no interaction with *SNR* [χ^2^(3)=0.9, p=0.832]. Similarly for PPD latency, there was a main effect of *response type* [χ^2^(1)=10.3, p=0.001], but no interaction with *SNR* [χ^2^(3)=1.3, p=0.730]. These findings confirmed that PPD increased in size and occurred later when sentences were not decoded perfectly (Fig.S2, top). Interestingly, this relationship held across subjects as well [r^2^=0.25, p=0.021, although one particular subject was a main contributor to this correlation], i.e. subjects who performed this task with greater difficulty exhibited larger PPDs than subjects who performed this task with ease. In contrast, there was no relationship between intelligibility and baseline [r^2^<0.01, p=0.805] confirming that PPD is the primary metric to look for, as long as participants engage in a single (and simple) task. This last statement may no longer hold when participants engage in multiple cognitive tasks [18].


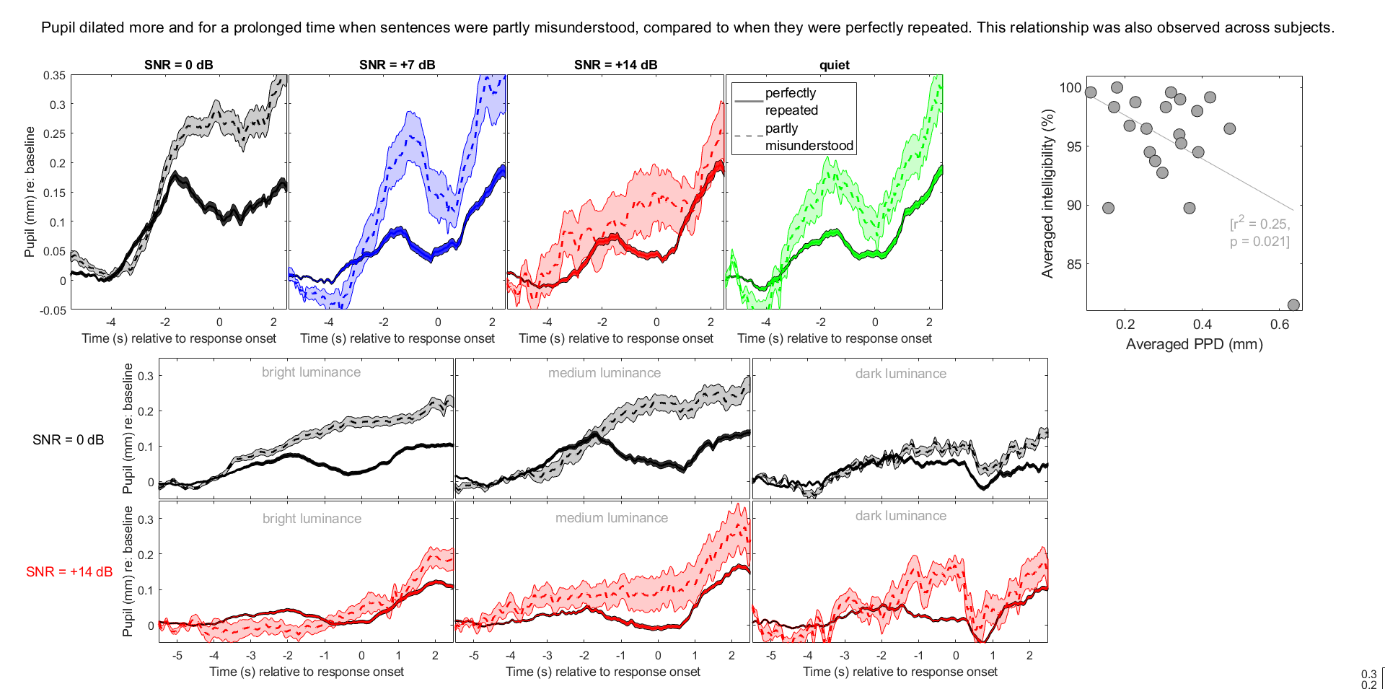


**S2 Appendix Fig. Baseline-corrected pupil traces in Exp.1 (top) and Exp.2 (bottom), as a function of whether the sentence was correctly recognized or partly missed.** PPDs were systematically larger and occurred later when sentences were misunderstood, and this pattern was common across luminance settings. Seen across individuals (top-right), PPD was also larger for the participants who struggled the most.

In Exp2, for PPD amplitude, there was a main effect of *response type* [χ^2^(1)=19.0, p<0.001], but it did not interact with *SNR* [χ^2^(1)=1.6, p=0.205], with *luminance* [χ^2^(2)=4.4, p=0.113], or in a 3-way [χ^2^(2)=1.5, p=0.484]. Similarly for PPD latency, there was a main effect of *response type* [χ^2^(1)=34.8, p<0.001], but it did not interact with *SNR* [χ^2^(1)<0.1, p=0.790], with *luminance* [χ^2^(2)=0.7, p=0.696], or in a 3-way [χ^2^(2)=3.6, p=0.168]. In other words, there was no evidence in the current data that differences in luminance changed anything to the behavior-to-pupillometry relationship: larger and later PPDs may be observed for misunderstood sentences even in bright or dark luminance, and regardless of SNR (Fig.S2, bottom). This is a very reassuring outcome with regard to past studies who might have recorded pupils under different behavioral manipulations, sometimes in suboptimal settings and their results would apparently still be valid.
