# Supplementary material for: Luminance effects on pupil dilation in speech-in-noise recognition
Source: PLoS One. 2022 Dec 2;17(12):e0278506. doi: 10.1371/journal.pone.0278506 (PMC9718387; doi:10.1371/journal.pone.0278506)
Supplement: Attachment — Submitted filename: rebuttal_letter .docx [file pone.0278506.s004.docx]

Reviewer #1: This manuscript describes a study to the effect of luminance and SNR on the peak pupil response dilation. Although the study methods are sound, the embedding of the study in the context of existing study can be improved substantially. Please find my suggestions below.
*1. Embedding in existing literature. The authors indicate that the study described in the manuscript is one of few studies addressing the combined influence of luminance and (auditory) task load on the pupil response (e.g. line 562). However, the authors did not include a rigorous description of current work. Specifically, the authors should include the studies cited below (and probably more relevant studies can easily be found) to provide a more comprehensive overview of the current state of knowledge. The current studies up to date suggest that the current results are inconclusive, namely some studies have found evidence for an effect of luminance on the PPD evoked by cognitive tasks, and some did not show this effect. The authors could expand the discussion about the factors that may be associated with this current inconclusive evidence.*

We thank our reviewers and academic editor to point us to all the relevant literature. We realized that many papers suggested are very helpful to improve the overall quality of our paper. We have included these studies in the introduction and discussion to improve the review of past studies (line88) and possible reasons that lead to inconsistencies in the existing studies (line114-122, line411 -430, line440).

*2. Currently, most published “guidelines” on pupillometry recommend to use a medium illumination level. How would the current results affect this recommendation? Importantly, the authors report a main effect of luminance, and the post-hoc analysis shows that the pupil response in bright conditions differs from that in medium level conditions. However, the difference between the dark and medium level condition is not significant. Regardless, the remaining part of the manuscript treats this absent effect as if this difference was indeed significant (e.g. line 395). Please adapt (why would you test the main effect if you don’t follow up on the results appropriately?).*

We agree with the reviewer that the main effect of luminance is not thoroughly followed up, therefore, we added extensive discussion on the results and why some results are different from past studies (i.e., medium luminance is bigger than both dark and bright) (line411-430). From our current results, in practice medium-luminance is still recommended (line591), and we added a note on how this might not be realistic in clinical settings with current setups and how this could be improved.

*3. Related to the incomplete account of the current literature, the authors do not explain and discuss the potential physiological mechanisms that may account for (part of) the effects. Specifically, although Steinhauer’s work is cited, they do not discuss the notion that the pupil response in darkness might be affected by a limited effect of the PNS on the pupil size in dark. Please include a discussion on the separate roles of PNS and SNS in the pupil response (and the effect of illumination)*

Indeed, we had not discussed the ANS mechanism sufficiently. We added a section in discussion to better explain/interpret our results (line411-440).

*4.* The authors suggest that luminance effects on the pupil response to cognitive load may be a negative aspect, and that it may “hinder” (e.g., line 82, line 120) the pupillometry applications. Rather, such effects can be exploited in case these are meaningful (e.g. Steinhauers work). Please discuss the potential benefits of such approaches.

Indeed, the complex relation between SNS and PNS can be experimentally controlled in a meaningful way (e.g., Steinhauer et al., 2004; Reilly et al., 2019) to answer interesting research questions. We have changed the tone when addressing the impact of luminance to be more neutral and added a note on how we can use the knowledge to benefit scientific research (line430). But we still keep and emphasize the central message of our paper that in the case of standardizing clinical pupillometry, factors like luminance and individual differences should be properly examined and controlled across clinics, in order to extract the pupillary responses to clinical stimuli of interest (i.e., standardized test words or sentences, pure tone or FM/AM tone for audiometry, visual stimuli etc) (line580). We think this message is more important (and also much-needed) for the field of cognitive hearing science.

*5. Line 68: a larger effect does not automatically indicate a predominant effect. Please rephrase.*

Thanks, we have rephrased ‘predominant’ to ‘having a larger impact’

*6. Line 136 and line 386: treating the baseline as being associated with the “tonic LC state” is probably not appropriate in the current study, as the baseline was derived just before the actual sentence presentation. The tonic LC state is observed during rest. The participants are not passively listening but probably actively anticipating the target speech. See also Joshi, S., & Gold, J. I. (2020). Pupil size as a window on neural substrates of cognition. Trends in cognitive sciences, 24(6), 466-480.*
Absolutely, thank you for pointing this out to us. We have removed the tonic/phasic state, and clarified that our baseline is not resting state baseline (line472).

*7. Please provide the rationale and hypotheses for the (additional) analyses performed. Please also provide the rationale for the specific analyses performed on the behavioural data. Are these analyses robust against the fact that the behavioural performances were at ceiling levels?*

We have added a section head to better explain our rationale for performing additional analyses (line 451). We also pointed more often to the additional analyses in the method and discussion section to support the value of the additional analyses (line 265 line 274, line410, line581). We believe that this will make the additional analyses section more relevant and will tie better with our central message. Behavioural results were indeed close to ceiling, as intended, due to participants being normally hearing and relatively young. The logistic mixed effect model does take into account such upper asymptote in performance and address the contrast better than applying transformations like arcsine-square-root (line212).

*8. The sentence duration needs to be provided in the main methods section. How is the varying sentence length accounted for with respect to the interval in which the pupil data were analysed? Was the interval adapted relative to the length of the shortest sentence? The additional analyses do not support this.*

The sentences remained unchanged and the analytical window contained sentence duration plus 2s of fixed waiting interval starting at the offset of the sentence for the pupil peak to emerge. We added further clarification to this point in line270. The uncorrected sentence duration is common in past listening effort studies because standardized sentences are almost always of slight duration difference (Winn et al., 2018, page 19). We also reported the 10^th^, 30^th^, 50^th^, 70^th^ and 90^th^ percentile of the distribution of all sentences (line 509). This distribution could give a better indication of the sentence duration. This duration variability does not affect averaged pupil response, as long as the time alignment of traces is done consistently (either by the onset or the offset of the sentence). We reported in the additional analyses the results of doing both types of alignment to ensure readers of our methods and to explore whether different alignment would change results significantly. A formal analysis was also performed to examine systematically whether variability in sentence duration, albeit small, has any impact on calculating task-evoked pupillary response.

*9. In many studies, the dynamic range of the pupil size that is used in the proportional baseline correction is specifically and separately measured (not during the actual task). This differs from the current approach. Could this have affected the results?*

We agree that resting state baseline would change the dynamic range calculation and baseline-corrected pupil response. And such baseline could also be affected by luminance. Considering the arousal that would take place when getting ready for a task, we further speculate that the resting state baseline would be lower than that we considered (prior to a given block, or across a whole experiment). In other words, the dynamic range we showed is presumably underestimated, compared to a method which would take the resting-state baseline instead. But note that other phenomena could also affect resting baseline other than task readiness. In our protocol, we did not include resting state pupil recordings, therefore, it is difficult for us to make any informed claim in this regard. We clarified that our baseline is not a resting state baseline and that we could get different results if we had done so. (line472).

*10. The resolution of the figures is poor, also when downloading the images directly from the portal.*

We have addressed this issue and uploaded images of correct resolution.

*11. The authors already interpret the data in the results section (e.g. lines 374-375). In addition, the authors could use more formal language throughout the paper (e.g. line 566: what is meant with “gripping”?).*

We have removed the data interpretation in the results section, except for one short sentence where we wish to highlight the key finding of the paper (line384). Also, we have improved the formality of the language to avoid using ‘gripping’ or other ambiguous words.

*12. Line 409: serperation typo*

The typo is now corrected.

*13. Lines 407-409: this is speculative; please indicate that this is not based on the current evidence.*
We agree; it is indeed a speculation based on the shape we obtained from the three luminance levels tested. We added possible support from the past literature (line 440), but neither of them provides direct support for this speculation, due to different test materials (Pan et al., 2022) and physiological biomarkers used (Slade et al., 2021). We have rephrased the sentence to highlight that this was only a speculation and more studies are needed to consolidate it.

Reviewer #2: This well-written and interesting work highlights a question of methodological significance in pupillometry (i.e., how should we account for variable lighting conditions?). The study was conducted with admirable rigor, and the data will contribute to our evolving understanding of how to optimize pupillometry and control for the all-important factor of luminance.
Thank you for your kind words. We’re glad you found it interesting.

This reviewer had several questions that the authors might wish to consider prior to this work:

One of the central claims of this work is that little is known about the interaction between task difficulty and luminance that might inform best practices in baseline correction. I would argue on this point that this is not entirely correct. The authors have missed several studies examining the effects of luminance perturbations on the magnitude of the task-evoked pupillary response function (Pan et al., 2022; Reilly et al., 2018). In addition, there are other past studies uncited (e.g., Bradhshaw, 1969) that involved similar manipulations from a half century ago. It is reasonably well-accepted that linear (subtraction) is the most biologically plausible form of baseline pupil correction (as referenced by Mathot et al). It might be useful to make contact with some of the following sources on the effects of luminance:
Bradshaw, J. L. (1969). Background light intensity and the pupillary response in a reaction time task. Psychonomic Science, 14(6), 271 -272.  <https://doi.org/10.3758/BF03329118>
Pan, J., Klímová, M., McGuire, J. T., & Ling, S. (2022). Arousal-based pupil modulation is dictated by luminance. Scientific Reports, 12(1), 1390. [https://doi.org/10.1038/s41598-022-05280-1](https://doi.org/10.1038/s41598-022-05280-1)
Reilly, J., Kelly, A., Kim, S. H., Jett, S., & Zuckerman, B. (2018). The human task-evoked pupillary response function is linear: Implications for baseline response scaling in pupillometry. Behavior Research Methods, 1–14.

We are incredibly grateful for this insight and the many suggestions on the literature. We have integrated these papers in the introduction and discussion to improve the coverage of past studies (line88) and possible reasons that could have led to inconsistencies in the existing studies (line114-122, line411 -430, line440).

*Pg. 8, I do not know what ‘IEEE sentences’ are– consider writing out acronyms and providing accompanying references
Pg. 8, I do not know what ‘HINT sentences’ are – consider writing out acronyms and providing accompanying references*

Thanks for pointing out the acronym issue. We have added the corpus name and the reference to the materials.

*Packages such as 'GazeR' implement velocity-based blink detection and interpolation procedures with the idea that observations corresponding to partial eyelid closure are part of the blink. It is not clear how/why you implemented your particular choice of blink correction algorithms. Consider elaborating*

Thank you for making us aware of these packages. At the time of the experimentation and data analysis, GazeR was not published (Apr 2020) so it did not come to our attention. Instead, we followed pupillometry guidelines provided in Winn et al., 2008 and Mathôt et al., 2018 when constructing pupil pre-processing pipeline. We reported in the Methods section the detailed steps and corresponding Matlab and R functions for analysis to promote reproducibility. Note that we also integrated a gaze-based exclusion (line233) rule, different from GazeR. We believe this addition could improve data quality, especially in settings like ours where participants looked at a very big screen and gaze wander-offs were common (easier than they might have been in another study with a typical laptop screen). Removing pupil measures with gaze too far away from the fixation cross can effectively control for the inattentive moments.

*It is not clear how you were able to obtain reliable pupil readings for the low (0 Lux) light condition. In our own past studies using luminance manipulations, we struggled to acquire reliable data in ‘pure’ dark even using an IR sensor with an Eyelink 1000. The eyetracker simply could not reliably discriminate pupil from iris in complete darkness. How did you shield the room from monitor luminance, etc. to achieve 0 Lux luminance?*

Most likely, this is because it wasn’t exactly 0 lux. Please have another read at the experimental room setup (line184), and you can find an actual photo of the setup below. The participants sat on a chair 2m away from a large screen (35-inch). In the dark condition, the room lights were turned off, and the screen luminance was set to the lowest for signaling participants. The room was indeed dark, but still with some dots of light emitted from the big screen. When we measured luminance using the luxometer TES-1335 at participants’ eye level 2m away from the screen, the reading was zero due to low screen light, big distance from the light source and possibly the sensitivity of the luxometer. But we acknowledge that we were not shielding all the light in the room, and agree with the reviewer that ‘complete darkness’ is not scientifically rigid. So, we have changed all occurrences of wording ‘complete darkness’ to ‘darkness’ or ‘close to 0lux’.
