# Supplementary material for: Luminance effects on pupil dilation in speech-in-noise recognition
Source: PLoS One. 2022 Dec 2;17(12):e0278506. doi: 10.1371/journal.pone.0278506 (PMC9718387; doi:10.1371/journal.pone.0278506)
Supplement: Attachment — Submitted filename: rebuttal_letter.docx [file pone.0278506.s005.docx]

We thank the two reviewers and the academic editor for their constructive comments which helped us improve this manuscript. We have made several edits to further polish this submission.

We apologize for the double edits in the earlier revision and fixed it in this new version to avoid confusion.

Note that line numbers are referenced to the clean version of this revision (not with tracked changes).

Reviewer #1: Review of PONE-D-22-07841_R1 “Luminance effects on pupil dilation in speech-in-noise recognition”.

The authors have improved the readability of the manuscript and better acknowledge the current state of literature in the introduction and discussion sections. I still have a few comments and questions:

Readability: The manuscript still contains quite a few typos and non-optimal phrases. I list a couple of them below, but there are probably more. Please carefully check the quality of the English text. Examples: line 46 “bigger and bigger”; line 71: this sentence doesn’t read fluently; line 75: “dark lighting” is a bit confusing, line 84: “it”: not clear what it refers to; line 105: “staining”, line 136: “they” should be there, line 140: “on” is missing, line 236: “as” should be and, line 256: add “rate”, line 296: this line refers to a result that hasn’t been presented yet, which is confusing; line 337: “our end goal” is not a clear phrase; line 339: sentence is not fluent, line 346: “disregarded” does not fit in the sentence; line 352: “massive”: informal language; which also holds for “little larger” in the next line.
We thank the reviewer to point out those non-optimal wordings and typos. We have made corresponding changes and also additional checks for typos (line 34, line 46, line71, line84, line105, line140, line 236, line256, line 296, line334, line337, line346,line352, line355 ).

Language: The authors describe that two types of sentences were used, either IEEE or HINT, depending on the native tongue of the participant. Please describe the characteristics of these sentence sets in more detail, as (differences in) complexity, sentence structure, sentence length etc. can all affect the performance and pupil response. This is especially relevant in relation to the analysis of the effect of sentence duration (was sentence set confounding, i.e. was the distribution of sentence duration the same for both sets?) and the determination of the analysis window. The analysis would benefit from an explicit comparison of the effect of sentence set / language to check whether it affected the dependent measures (even though the limited sample size in such a comparison probably reduces the chances of observing an effect). Line 299 and line 528: Please explain what is meant with “with different speech materials”? Did the listeners perceive another (third) set of sentences? Please describe the details.
We thank the reviewer for requesting the precision of the sentences used. We added clarification in the section of method and additional analysis that the sentence distribution calculation is done on all the sentences, and the analysis window is from the sentence onset to the repeat prompt (line 291, line511). We also clarified in line229 and line528 that the ‘different’ materials referred to sentence not used in the previous experiment.

We agree that the HINT and the IEEE materials are different, not only in their length but also their semantic complexity. The analysis presented in Additional analysis deals to some extent with this variable since effects of duration are pooled together across the 2 materials, so there was more representation of French sentences in the long and very-long bins and more representation of English sentences in the short and very-short bins. However, we do not wish to draw the reader's attention to this language-induced difference because it is not a factor of interest in our scientific hypothesis, and it is not clear that the effect of language applies differentially to French and English participants. In other words, French participants may be used to relatively longer sentences than English participants (because of the way each language is constructed). So, the effect of duration might apply over different ranges in each language. This speculation is something for future empirical studies to resolve. We do not have the statistical power in this study to examine it properly, considering the number of main effect factors we are already investigating. What we ensured in the experimental design is that each participant performed the task in their native language, hence no extra cognitive effort required for processing non-native language. In this regard, the language of the materials was irrelevant. None of the present findings are about cognitive load due to lack of mastery in the materials. Therefore, effects of duration (and necessarily materials) have been postponed to the very end of the discussion (last section of Additional analyses) to make sure that the reader focuses on the take-home message of the paper which is about light effect and their interaction with SNR.

Line 311: Why do the authors refer to the current task as a “non-demanding” task? Please explain.

Speech in noise tasks conducted between 0 and 14 dB SNR are generally not considered difficult for NH listeners. The speech intelligibility performance is high (>90%) and approaching ceiling (line 292). The wording of “non-demanding” is consistent with previous literature in NH listeners.

Figure 2: to me, it is not clear why the pupil response in quiet, which seems relatively large in Figure 2 (plot showing the response over time) is actually relatively small in the plot showing mean PPD. What is causing the relative difference between the size of the effect between these plots? How do the authors explain the relatively large pupil response in the most easy condition?

This difference is due to the inherent variability in the individual pupil traces. When averaging traces who peak at slightly different times, the resulting average is smoothed down and could have a peak latency that is also slightly different from the average of the individual peak latencies. That is true of pupillometry just like many other neurophysiological techniques.  For the PPDs shown in the errorbar plots and in statistical analysis, we calculate one PPD from aggregated 20 traces in a list, and then those more stereotypical traces are entered into statistics and calculating the Grand-average trace.This is similar to the difference when we calculate PPD based on single trial and based on list aggregation (PPD of a trace aggregated from 20 traces), in Supplementary materials.

In response to the latter question, the quiet condition did not differ significantly from +14 or even +7 dB; it’s really at 0 dB SNR that PPD amplitude and latency started to differ. So, we didn’t delve into this speculation.

Absent interaction between SNR and luminance: none of the many analyses found an interaction effect between SNR and luminance. However, the wording of some sentences is a bit confusing and seems to refer to such an interaction. For example: line 392: “interact”: please rephrase.

This is essentially because we suspect the interaction to exist but could not demonstrate it. For sake of rigor, we clarified (start of the second paragraph of the discussion of Exp2) that: “The lack of significant interaction between luminance and SNR conditions in our results show that this bias is relatively consistent across SNRs”. However, in the rest of this paragraph, we did delve into the speculation that an interaction might exist given a more global understanding of the pattern in our data as well as the existing literature.

Discussion: The way in which additional analyses and results are reported in the discussion section is not usual and not introduced properly. Consider moving these sections to the results section.

We have considered the re-organisation. However, the current format best suits the logic of the paper. The result section directly reflects our answers to the initial hypothesis. Then upon checking the results, further analyses were required in order to better understand possible reasons to explain our rather surprising results, i.e., we did not expect the effect of luminance to be of such a big impact on task-evoked response. To move the additional analysis to the results section might confuse the readers with the central message of our article and the flow of scientific hypothesis testing.

Reviewer #2: The authors have addressed many of this reviewer's original concerns but might consider the following issues:
1) Effect size measurements should appear in conjunction with relevant statistics to promote replication (mean diff, pooled std of the groups).

We thank the reviewer for pointing this out. We have added in the results section group mean and std where relevant (line289, line325, line 384, line391, line425)

2) It is unclear why the authors chose to implement proportional baseline scaling. This technique is not recommended as a baseline correction procedure as it can significantly distort the magnitude of evoked pupil dilation at high/low levels of the dynamic range. For example, an evoked change of .1mm from a 1mm baseline is a much higher %change relative to the same absolute change (.1mm) from a 9mm baseline. Several recent papers have addressed this issue along with recommendations for subtractive baseline scaling.

We did not choose proportional baseline scaling but a subtractive method. This was exactly the point of one section in Additional Analysis Effect of PPD calculation methods. In the main results reporting section (line263), we used subtractive method exactly due to the reasons proposed by the reviewer.

3) It is reasonably well established that at very high ends of the dynamic range of the pupil (e.g., intense light, absolute darkness), pupillary movements become idiosyncratic. In addition, eye trackers often become unreliable in these conditions because of challenges in contrasting pupil from iris. It was not clear what the overarching recommendations were here regarding control for luminance. In moderate ambient lighting conditions as most labs might encounter (e.g., fluorescent lighting) practical variability would not be too high (assuming testing in a windowless room).

We agree with the reviewer that in summary we recommend moderate ambient lighting for pupillometry experiments (line596). Our paper serves as a scientific investigation of how in low and high light, the effect of light is beyond just physiological constraints, but also with the task-evoked pupillary response. Previous studies acknowledged in the introduction had suggested that the relation between task-evoked and light-evoked pupillary response is complex and not completely consistent. Therefore, our study further contributes to this line of study to investigate this relation between two types of pupillary responses. We also made further analysis to explain the confounds of other possible factors in the experimental design that were not done in previous studies. Altogether, we believe that we presented a rigorous scientific case that luminance control is very important in pupillometry experiments and should be emphasized more in methods reports and experimental design.

4) I had difficulty finding links to a data repository to examine stimuli, etc.

To ease the access to data, we provided a zip file of all behavioural and pupil raw data with the submission. The zip file should appear at the end of the manuscript visible to the reviewer. However, we do not have the copyright to share the sentence stimuli (IEEE and HINT sentences) and they are only used in the experiment after the authorization of the owner and distributor of the materials.
